# Phenotypic and Molecular Characterization of Three Clinical Voriconazole-Resistant *Aspergillus fumigatus* Isolates Harboring the *cyp51A* TR46/Y121F/T289A Genotype

**DOI:** 10.3390/pathogens15101078

**Published:** 2026-10-13

**Authors:** Xuemei He, Yingyu Mao, Duxin Liu, Xiaofang Zheng, Kaibo Song, Fangyan Liu, Zhangyong Song

**Affiliations:** 1 School of Basic Medical Sciences, Southwest Medical University, Luzhou 646000, China; hxm1418@163.com (X.H.); tianya1000@126.com (Y.M.); dxliu2025@163.com (D.L.); zxflmbf@163.com (X.Z.); 13333786313@163.com (K.S.); 2 Molecular Biotechnology Platform, Public Center of Experimental Technology, Southwest Medical University, Luzhou 646000, China; 3 Hemodynamics and Medical Engineering Combination Key Laboratory of Luzhou, Southwest Medical University, Luzhou 646000, China

**Keywords:** *Aspergillus fumigatus*, voriconazole resistance, *cyp51A*, TR46/Y121F/T289A, ergosterol biosynthesis, efflux pump

## Abstract

Azole-resistant *Aspergillus fumigatus* poses an increasing clinical challenge. In this study, 22 clinical filamentous fungal isolates from southwestern China were preliminarily identified as *A. fumigatus* based on morphological characteristics, and their antifungal susceptibility was determined using broth microdilution. Three isolates (AF268, AF275 and AF277) exhibiting high-level voriconazole resistance were selected for further phenotypic and molecular characterization. Compared with AF293, the three resistant isolates showed no significant differences in biofilm metabolic activity or biomass, but all exhibited significantly reduced conidiation at 72 h. In a *Galleria mellonella* infection model, these isolates exhibited attenuated virulence, as indicated by increased larval survival and reduced fungal burdens. All three isolates harbored the *cyp51A* TR46/Y121F/T289A genotype and shared Hmg1 S212P/Y564F variants, while no Cyp51B substitutions were detected. RT-qPCR analysis further indicated transcriptional dysregulation of ergosterol biosynthesis- and efflux pump-associated genes. These findings show that the three clinical isolates carrying the canonical *cyp51A* TR46/Y121F/T289A resistance genotype showed differences in biological traits and transcriptional responses relative to the reference strain AF293. The high voriconazole MICs are consistent with the shared *cyp51A* genotype, whereas the functional significance of the Hmg1 variants and transcriptional changes requires further genetic validation.

## 1. Introduction

*Aspergillus fumigatus*, an opportunistic fungal pathogen widely distributed in the environment, is the most frequently isolated *Aspergillus* species in clinical settings and the major etiological agent of human aspergillosis [1,2,3]. Its asexual propagules (conidia) are dispersed through the air and enter the respiratory tract by inhalation [2]. In immunocompetent hosts, inhaled conidia are generally eliminated efficiently by the innate and adaptive immune responses. However, in immunocompromised individuals, inhaled conidia can colonize the respiratory tract, germinate into invasive hyphae, and invade tissues, leading to respiratory tract infection and, in severe cases, disseminated (systemic) infection [3,4]. *A. fumigatus*-associated aspergillosis comprises a broad spectrum of clinical phenotypes, with the major entities including allergic bronchopulmonary aspergillosis (ABPA), chronic pulmonary aspergillosis (CPA), and invasive aspergillosis (IA) [2,3,5]. IA is characterized by rapid progression and poor outcomes, and represents one of the most severe clinical forms of infection [6,7,8]. The estimated annual global incidence of IA is ~2.10 million cases [9]. Reported case-fatality rates among patients with chronic obstructive pulmonary disease range from ~43% to 72% and those of patients in intensive care units (ICUs) range from ~46% to 82% [9]. Estimates of the global burden suggest that >4.8 million people are affected by ABPA [10]. Modeled estimates indicate that CPA causes ~1.837 million new cases and ~340,000 deaths each year [9]. In recent years, the population susceptible to aspergillosis has continued to expand. This expanding at-risk population includes patients receiving transplantation, anticancer therapy, or immunomodulatory treatment, as well as critically ill patients with severe viral infections [3,11]. For example, invasive aspergillosis has been reported in up to approximately 38% of critically ill patients with severe viral infections, including COVID-19, with mortality exceeding 50% in some cohorts [11]. In 2022, the World Health Organization (WHO) listed *A. fumigatus* in the highest “critical priority” tier in its fungal priority pathogens report, underscoring its growing clinical and public health importance [12].

Mold-active triazoles and polyenes are major antifungal classes used in the treatment of aspergillosis [13,14]. Following a landmark randomized trial, voriconazole (VRC) became established as a first-line agent for invasive aspergillosis [15], and mold-active triazoles continue to play a central role in the treatment of invasive pulmonary aspergillosis [14]. Experimental immunomodulatory strategies have also been investigated, including pharmacological modulation of macrophage responses to *A. fumigatus* infection [16]. However, triazole resistance in *A. fumigatus* has become increasingly recognized and varies considerably across geographic regions and clinical centers [11,17]. Major resistance mechanisms include alterations in the azole target *cyp51A* [18,19], transcriptional upregulation of drug-efflux systems [20], and perturbation of sterol homeostasis, including changes involving *hmg1* [21,22]. Broader transcriptional responses to azole exposure may also contribute to fungal adaptation under drug pressure [23]. Environmental exposure to agricultural azole fungicides may select for resistant genotypes with cross-resistance to medical triazoles [19,24]. Among *cyp51A*-mediated mechanisms, promoter tandem repeats combined with coding-region substitutions are particularly important. TR34/L98H and TR46/Y121F/T289A are well-established genotypes associated with environmentally selected azole resistance [18,19,24]. The TR46/Y121F/T289A genotype is particularly associated with reduced susceptibility to VRC, although susceptibility to other azoles may vary among isolates [25]. These findings indicate that the *cyp51A* genotype alone does not fully determine the azole susceptibility profile of an isolate. Additional resistance-associated mechanisms, including alterations in sterol homeostasis and strain-specific regulation of efflux pathways, may further influence the observed phenotype [20,22,23]. Recent surveillance in Ningxia reported an overall azole-resistance rate of approximately 1.20%, further highlighting regional variation within China [26]. Our group has previously investigated the biological characteristics and mechanisms of azole resistance in clinical *A. fumigatus* isolates [27].

In the present study, we characterized three clinical *A. fumigatus* isolates with high-level VRC resistance selected from 22 clinical isolates preliminarily identified as *A. fumigatus* collected in southwestern China. We focused on their antifungal susceptibility, biological phenotypes, virulence, and resistance-associated molecular features, with each isolate evaluated relative to the reference strain AF293 despite their shared *cyp51A* TR46/Y121F/T289A genotype.

## 2. Materials and Methods

### 2.1. Strains and Culture Methods

A total of 22 clinical isolates were included in this study. All isolates were obtained from a strain collection and were kindly provided by a tertiary (Grade A, Class III) hospital in southwest China. Patient information is summarized in Supplementary Table S1. The isolates were cultured on potato dextrose agar (PDA) supplemented with penicillin–streptomycin at 37 °C for 5 days. Preliminary identification was made based on the macroscopic colony morphology and microscopic characteristics [27]. The reference strain was *A. fumigatus* strain AF293. All strains were stored in glycerol stocks at −80 °C. Before each experiment, the strains were sub-cultured on PDA at 37 °C for 5–7 days. Conidia were harvested by filtration through 50 µm mesh and suspended in sterile water containing 0.05% Tween 80. The conidial concentration was determined with a hemocytometer and the suspension was used as the stock inoculum [27].

### 2.2. Antifungal Susceptibility Testing

Antifungal susceptibility testing was performed with the broth microdilution method for filamentous fungi, in accordance with the Clinical and Laboratory Standards Institute (CLSI) guideline CLSI M38 [28]. Briefly, antifungal powders were dissolved in dimethyl sulfoxide to prepare stock solutions at 5.12 mg/mL. For the initial susceptibility screening of the 22 clinical isolates, the stocks were diluted in RPMI 1640 to obtain the following final concentration ranges: amphotericin B (AMB), 32–0.25 µg/mL; fluconazole (FLC), 128–2.0 µg/mL; voriconazole (VRC), 32–0.25 µg/mL; and itraconazole (ITR), 32–0.25 µg/mL. For further susceptibility testing of AF268, AF275, AF277, and the reference strain AF293, the stocks were diluted in RPMI 1640 to obtain the following final concentration ranges: micafungin (MF), 4–0.03125 µg/mL; anidulafungin (ANI), 4–0.03125 µg/mL; posaconazole (POS), 16–0.25 µg/mL; and isavuconazole (ISA), 16–0.25 µg/mL. Conidial suspension (1.0 × 10^5^ cells/mL) was dispensed into 96-well microtiter plates and incubated at 37 °C for 48 h. Wells with no drug were used as the growth controls. MIC was defined as the lowest drug concentration that resulted in the complete inhibition of visible growth after 48 h at 37 °C. The minimum effective concentration (MEC) was defined as the lowest drug concentration that induced short, thick hyphae, assessed by microscopic examination [28,29].

The agar disk diffusion assay was conducted based on a previously described protocol, with minor modifications [30]. Briefly, 100 µL of conidial suspension (1.0 × 10^6^ conidia/mL) was evenly spread on RPMI 1640 agar with a sterile cotton swab. After the plates were dried for 15–30 min, antifungal disks (Liofilchem S.r.l., Roseto degli Abruzzi, Italy) were placed at the centers of the agar surfaces with sterile forceps. The plates were incubated at 37 °C for 48 h, and the zones of inhibition were recorded.

### 2.3. Morphological and Molecular Identification, Sequence Analysis, and Colony Growth Assessment

Strains AF268, AF275, and AF277, and reference strain AF293 were cultured on PDA at 37 °C for 5 days. A small amount of colony material was collected and stained with lactophenol cotton blue. The conidial and hyphal morphologies were examined under a microscope.

To extract genomic DNA, RPMI-1640 medium was inoculated with conidia from each strain and incubated at 37 °C with shaking at 200 rpm for 48 h. The mycelia were harvested and ground to a fine powder in liquid nitrogen. *A. fumigatus* genomic DNA was extracted with a commercial DNA extraction kit (Beijing Kangwei Century Biotechnology Co., Ltd., Beijing, China). PCR was used to amplify the internal transcribed spacer (*ITS*) region, β-tubulin (*benA*) locus, and the *cyp51A*, *cyp51B*, and *hmg1* genes. The primer sequences are listed in Supplementary Table S2. The PCR products were subjected to Sanger sequencing. The ITS and β-tubulin sequences were aligned separately with corresponding reference sequences retrieved from NCBI Nucleotide [31]. Phylogenetic trees for each locus were constructed in MEGA11 using the neighbor-joining method with 1000 bootstrap replicates (MEGA, LLC, Baltimore, MD, USA).

To assess colony growth, conidial suspensions of AF268, AF275, AF277, and AF293 were prepared at 1 × 10^6^ conidia/mL, as described above. A 3 µL aliquot of each suspension was spotted onto the centers of PDA plates. The plates were incubated at 37 °C. Radial colony growth was measured daily to determine the growth rates [27].

### 2.4. Conidiation and Biofilm Assays

To quantify conidiation, conidial suspensions of AF268, AF275, AF277, and AF293 were prepared at 1 × 10^6^ conidia/mL, as described above. A 3 µL aliquot of each suspension was spotted onto the centers of PDA plates. After incubation for 72 or 120 h, all conidia were recovered by washing the colonies with sterile water containing 0.05% Tween 80. The suspensions were serially diluted, and the conidia were counted with a hemocytometer.

Biofilm formation was assessed with the 2,3-bis(2-methoxy-4-nitro-5-sulfophenyl)-5-[(phenylamino)carbonyl]-2H-tetrazolium hydroxide (XTT)–menadione assay [32] and the crystal violet (CV) assay [33]. Conidial suspension in RPMI-1640 medium (1.0 × 10^5^ cells/mL) was dispensed into 96-well tissue culture plates and incubated at 37 °C for 24 h. The medium was then removed, and the wells were washed three times with phosphate-buffered saline (PBS) to remove nonadherent conidia and hyphae. To measure the metabolic activity of the biofilm, the supernatant was removed as described above, and 200 µL of XTT sodium salt–menadione working solution was added to each well. The plates were incubated at 37 °C in the dark for 2 h. Then, 150 µL of the supernatant was transferred to a new 96-well plate, and the absorbance at a wavelength of 490 nm was measured with a microplate reader.

The biofilm biomass was quantified with CV staining. After the supernatant was removed, 100 µL of 0.1% CV was added to each well and incubated at room temperature for 15 min. The stain was removed, and the wells were washed three times with PBS. Then, 33% acetic acid was added to the wells and incubated for 45 min. Absorbance at a wavelength of 600 nm was measured with a microplate reader.

### 2.5. Virulence Analysis in Galleria mellonella

Healthy *G. mellonella* larvae weighing 200–300 mg and approximately 3 cm in length were used for the analysis. *A. fumigatus* conidia were suspended in sterile PBS and adjusted to 1 × 10^6^ conidia/mL. Each larva was inoculated with 10 µL of the suspension, with 10 larvae per group. After inoculation, the larvae were incubated at 37 °C in the dark. Mortality was assessed daily for 5 days. Death was defined as an absence of movement and evidence of melanization.

Survival was monitored and the fungal burden determined in independent groups of larvae. On days 1 and 3 post-infection, six larvae from each group were randomly selected and transferred to Eppendorf tubes. The larvae were homogenized in PBS and serially diluted. A 30 µL aliquot of each dilution was spread evenly onto PDA plates supplemented with ampicillin–penicillin. The plates were incubated at 37 °C for 24 h, and the colony-forming units were counted [34].

### 2.6. Quantitative Real-Time PCR (qPCR) Assay

The conidia were harvested from *A. fumigatus* colonies grown on PDA plates and used to inoculate RPMI-1640 medium (final concentration, 1.0 × 10^6^ cells/mL). The cultures were incubated at 37 °C with shaking at 200 rpm for 16 h. Voriconazole (16 µg/mL) was then added, and incubation was continued for a further 8 h. The cells were washed three times with PBS and collected by centrifugation. The pellets were ground repeatedly in liquid nitrogen, and the total RNA was extracted with RNAiso Plus (Takara Bio Inc., Shiga, Japan). The RNA concentration was measured, and cDNA was synthesized with the PrimeScript™ FAST RT Reagent Kit (Takara Bio Inc., Shiga, Japan). Target gene expression was quantified with a qPCR assay. The primer sequences are provided in Supplementary Table S2. β-Tubulin mRNA was used as the internal reference. Relative expression levels were calculated with the 2^−ΔΔCT^ method [35].

### 2.7. Statistical Analysis

Data were analyzed with GraphPad Prism (version 9.5; San Diego, CA, USA). For the conidiation, biofilm, and fungal-burden assays, each clinical isolate was compared with AF293 using one-way ANOVA followed by Dunnett’s multiple-comparisons test; different time points were analyzed separately. Survival curves for each clinical isolate were compared with AF293 using the log-rank (Mantel–Cox) test with Bonferroni correction for three prespecified comparisons. For the qPCR analyses, individual genes were compared using multiple unpaired two-tailed Student’s *t*-tests with Holm–Šidák correction for multiple comparisons within each panel. All experiments were performed independently in triplicate, unless otherwise stated. All statistical tests were two-sided, and *p* < 0.05 was considered statistically significant. Adjusted *p* values were used for analyses involving multiple comparisons.

## 3. Results

### 3.1. Clinical Isolation and Identification of Antifungal-Resistant Isolates

**Figure 1:**
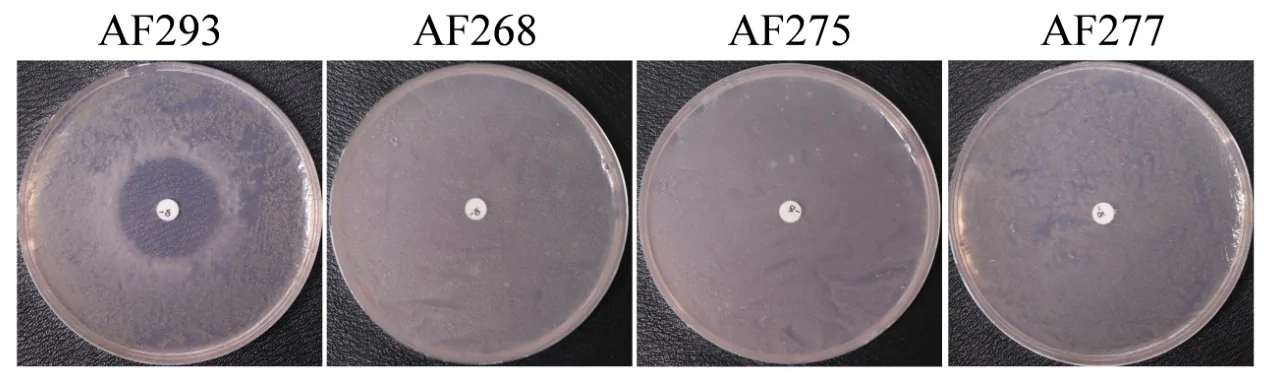
Screening the target strains with the disk diffusion susceptibility test (VRC, 1 µg). Plates were incubated at 37 °C for 48 h, and inhibition zones were recorded.

In total, 22 clinical filamentous fungal isolates were analyzed in this study. Their clinical characteristics are summarized in Supplementary Table S1. The patients from whom they were collected were 53–86 years old, and more than 80% were male (18/22). Most isolates were obtained from respiratory specimens, with sputum accounting for 86.4% (19/22). According to a CLSI broth microdilution analysis, three isolates (AF268, AF275, and AF277) showed VRC resistance, with MICs of 32 μg/mL (Table 1). Consistent with this, these isolates showed no inhibition zones in the 1 μg VRC disk diffusion assay, in contrast to the susceptible reference strain AF293 (Figure 1). Furthermore, AF268, AF275, and AF277 isolates were further tested for their susceptibility to other antifungal agents. In contrast to their high VRC MICs, AF268, AF275, and AF277 showed relatively low MICs for AMB, ITR, and POS and low MECs for the echinocandins tested, whereas their ISA MICs were >16 µg/mL, compared with 0.5 µg/mL for AF293 (Table 2).

### 3.2. Morphological and Molecular Identification of Isolates AF268, AF275, and AF277

**Figure 2:**
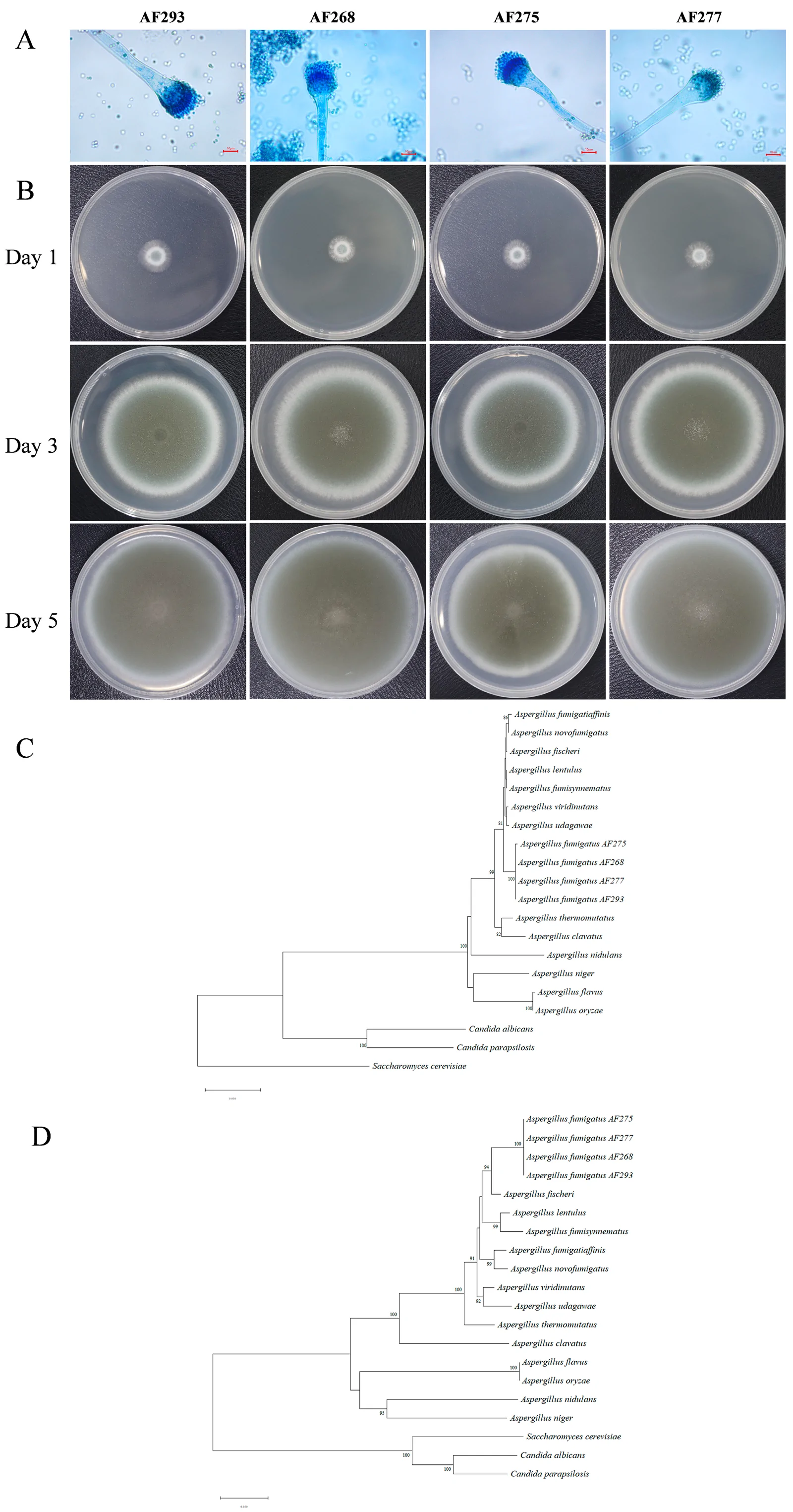
Morphological and molecular identification of isolates AF268, AF275, and AF277. (**A**) Microscopic morphology after lactophenol cotton blue staining. Scale bar, 10 μm. (**B**) Colony morphology of the tested strains on PDA at 37 °C. (**C**,**D**) Neighbor-joining phylogenetic trees based on ITS (**C**) and β-tubulin (**D**) sequences of AF268, AF275, AF277, the reference strain AF293, and reference sequences retrieved from NCBI Nucleotide. Trees were constructed in MEGA11 using p-distances. Numbers at nodes indicate bootstrap support (%) based on 1000 replicates. Scale bars indicate 0.05 nucleotide differences per site.

After lactophenol cotton blue staining, microscopic examination showed that strain AF293 and the clinical isolates AF268, AF275, and AF277 displayed the conidiophore and conidial head structures typical of *A. fumigatus* (Figure 2A). When cultured at 37 °C and monitored at 1, 3, and 5 days, all strains showed radial, floccose-to-velvety colony growth. The colony color gradually shifted from white to smoky gray or green. Notably, strain AF275 produced smaller colonies than the other strains on days 3 and 5 (Figure 2B). Both ITS and β-tubulin phylogenetic analyses placed AF268, AF275, and AF277 in the same clade as the reference strain AF293, with 100% bootstrap support (Figure 2C,D), supporting their identification as *A. fumigatus*.

### 3.3. Conidiation Capacity and Biofilm Characteristics of the Isolates

**Figure 3:**
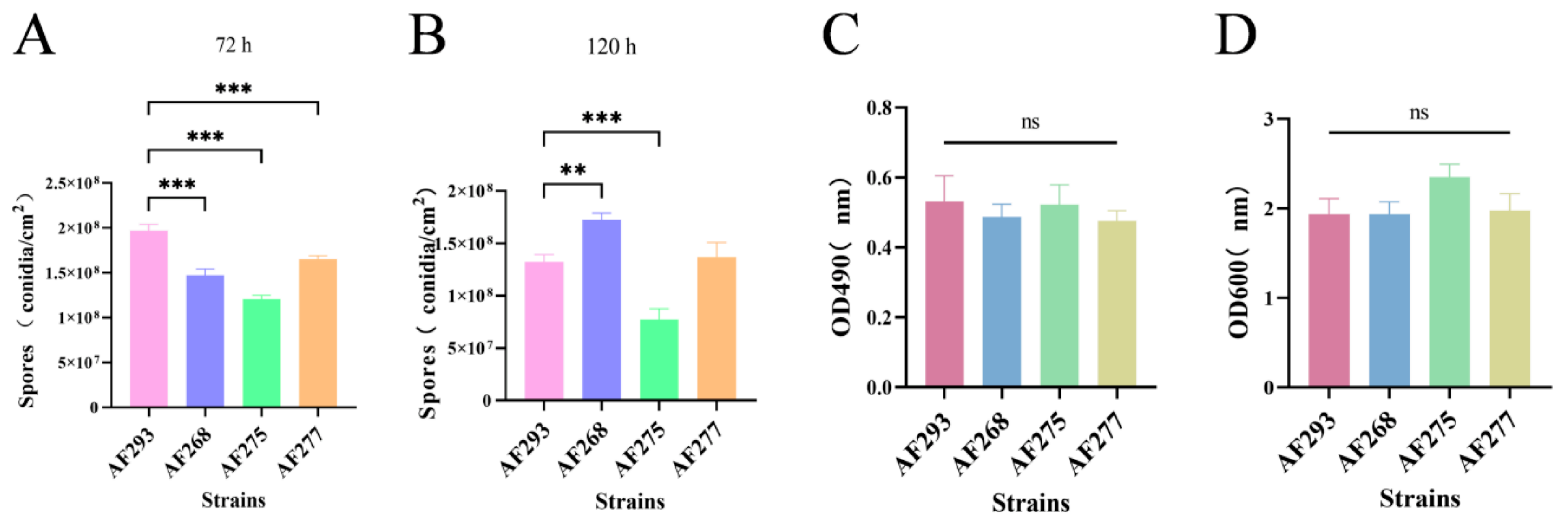
Comparison of conidiation capacity and biofilm characteristics. (**A**,**B**) Conidial counts of the four strains at 72 and 120 h, analyzed separately. (**C**) Biofilm metabolic activity measured using the XTT–menadione assay; absorbance was measured at 490 nm. (**D**) Biofilm biomass measured by crystal violet staining; absorbance was measured at 600 nm. Statistical comparisons were performed using one-way ANOVA followed by Dunnett’s multiple-comparisons test, with AF293 as the comparator. ns, not significant; ** *p* < 0.01, *** *p* < 0.001.

Conidial production was assessed at 72 and 120 h of incubation, with each clinical isolate compared individually with AF293 at each time point. At 72 h, strain AF293 produced significantly more conidia than did the three clinical isolates. At 120 h, strain AF268 produced significantly more conidia than strain AF293. In contrast, strain AF275 showed significantly reduced conidiation at both time points compared with that of strain AF293 (Figure 3A,B). None of the three clinical isolates differed significantly from AF293 in biofilm metabolic activity or biomass (Figure 3C,D).

### 3.4. Virulence Characteristics of the Test Strains

**Figure 4:**
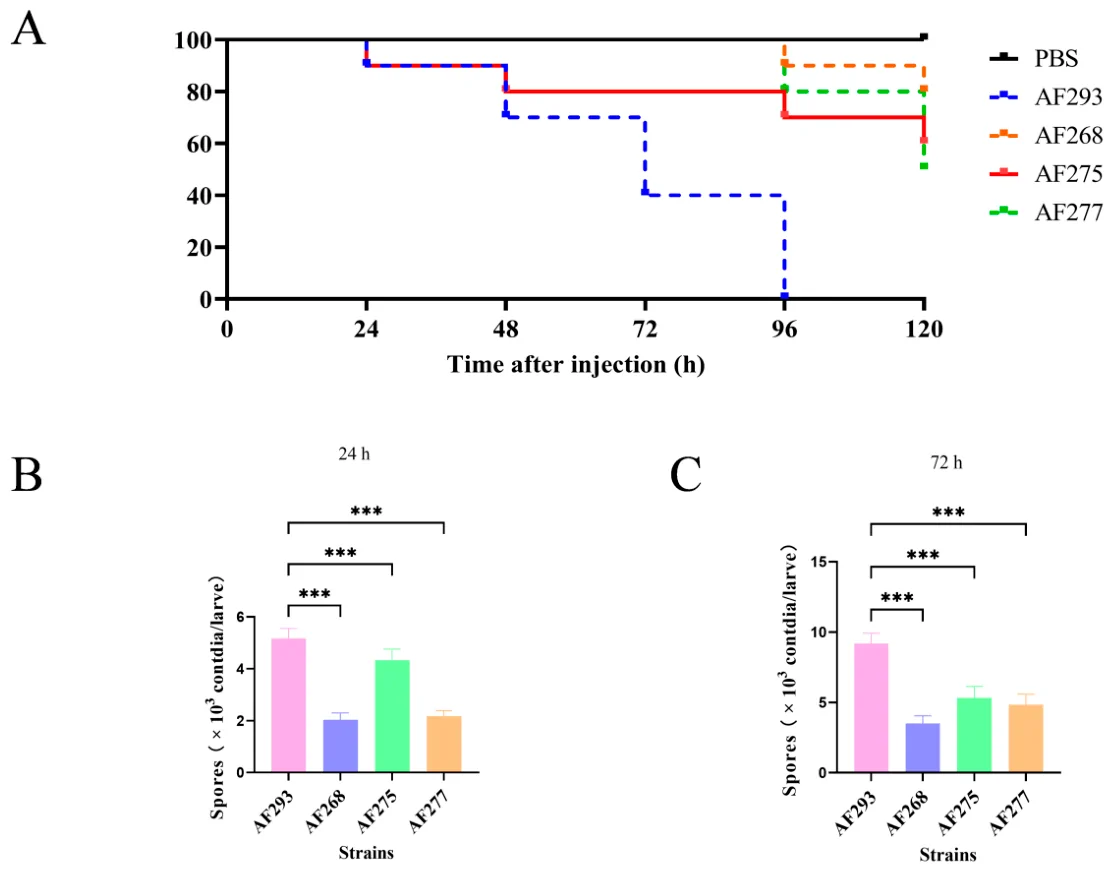
Comparison of survival and in vivo fungal burden in the *Galleria mellonella* infection model. (**A**) Survival curves for AF268, AF275, and AF277 were each compared with AF293 using the log-rank (Mantel–Cox) test with Bonferroni correction for three prespecified comparisons (AF268 vs. AF293, *p* < 0.001; AF275 vs. AF293, *p* = 0.015; AF277 vs. AF293, *p* < 0.001). (**B**,**C**) Fungal burdens at 24 and 72 h were analyzed separately using one-way ANOVA followed by Dunnett’s multiple-comparisons test, with AF293 as the comparator. *** *p* < 0. 001.

The virulence of each clinical isolate was compared with that of AF293 using the *G. mellonella* larval infection model. During the observation period, survival remained at 100% in the PBS-injected control group, indicating that the experimental procedure did not affect larval mortality. After infection with strain AF293, larval survival decreased markedly by 72 h, and all the larvae had died by 96 h. In contrast, larvae infected with clinical strains AF268, AF275, or AF277 showed significantly greater survival than those infected with AF293 (Figure 4A). Consistent with this, the fungal burden measured at 24 and 72 h post-infection was highest in the AF293 group. The fungal burden was significantly lower in the three clinical isolate groups than in the AF293 group (Figure 4B,C).

### 3.5. Sequence Analysis of cyp51A, cyp51B, and hmg1

**Figure 5:**
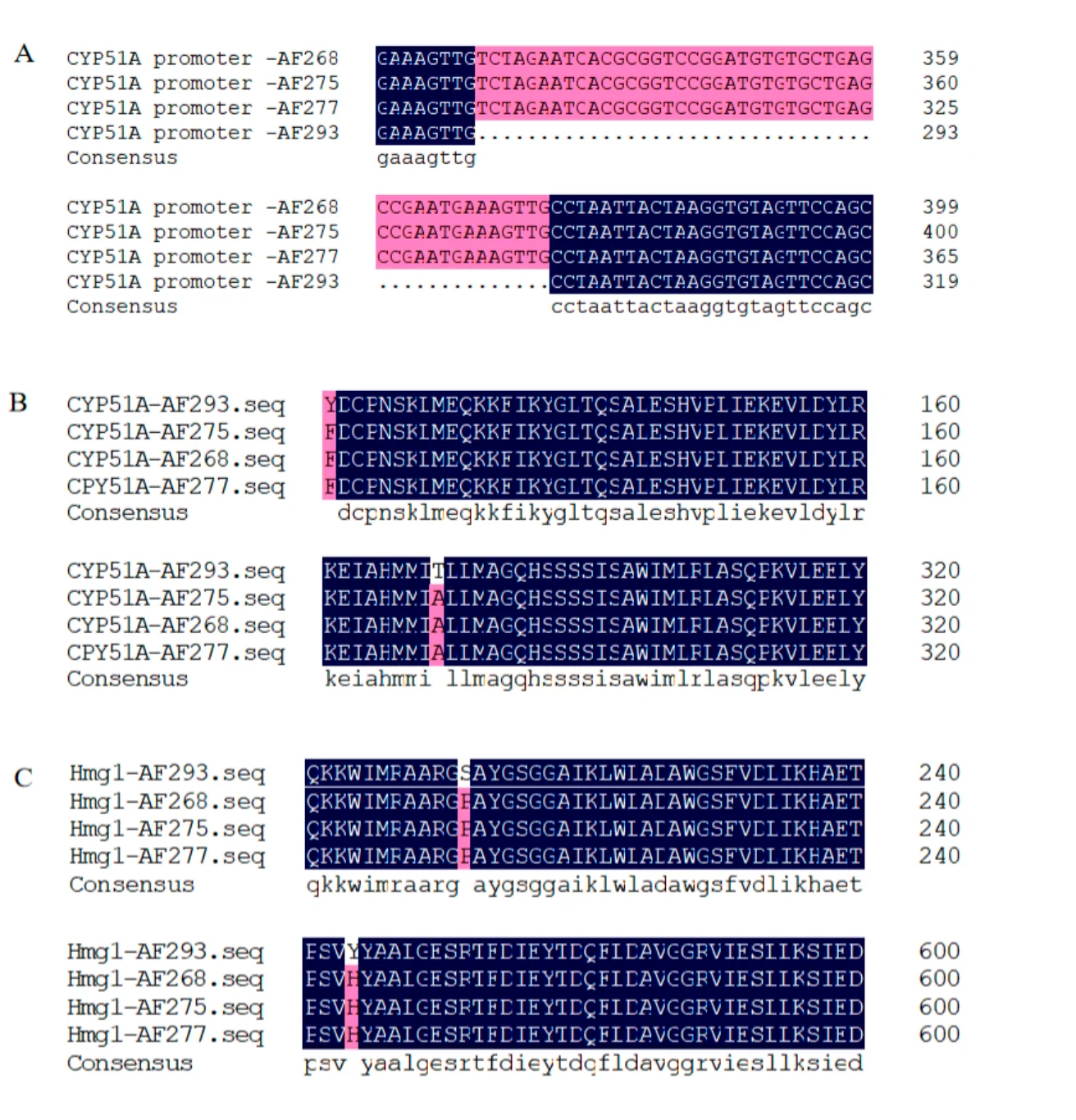
PCR amplification and sequence alignment analysis of the *cyp51A* promoter and the deduced Cyp51A and Hmg1 amino acid sequences in the tested *Aspergillus fumigatus* strains. (**A**) Alignment of the *cyp51A* promoter regions showing the 46 bp tandem repeat in AF268, AF275, and AF277 relative to AF293. (**B**) Alignment of the deduced Cyp51A amino acid sequences showing Y121F and T289A substitutions. (**C**) Alignment of the deduced Hmg1 amino acid sequences showing S212P and Y564F substitutions.

All three clinical isolates (AF268, AF275, and AF277) carried a 46 bp tandem repeat in the *cyp51A* promoter (TR46), together with the Cyp51A amino acid substitutions Y121F and T289A (Figure 5A,B). Additional Cyp51A amino acid differences, including Y46F, V172M, T248N, E255D, and K427E relative to AF293, were detected in all three clinical isolates and are shown in Supplementary Figure S1. No amino acid substitutions were detected in Cyp51B in any of the three clinical isolates compared with AF293. All three clinical isolates also carried the Hmg1 amino acid variants S212P and Y564F (Figure 5C).

### 3.6. Expression of Ergosterol Pathway and Efflux Pump Genes Before and After VRC Treatment

**Figure 6:**
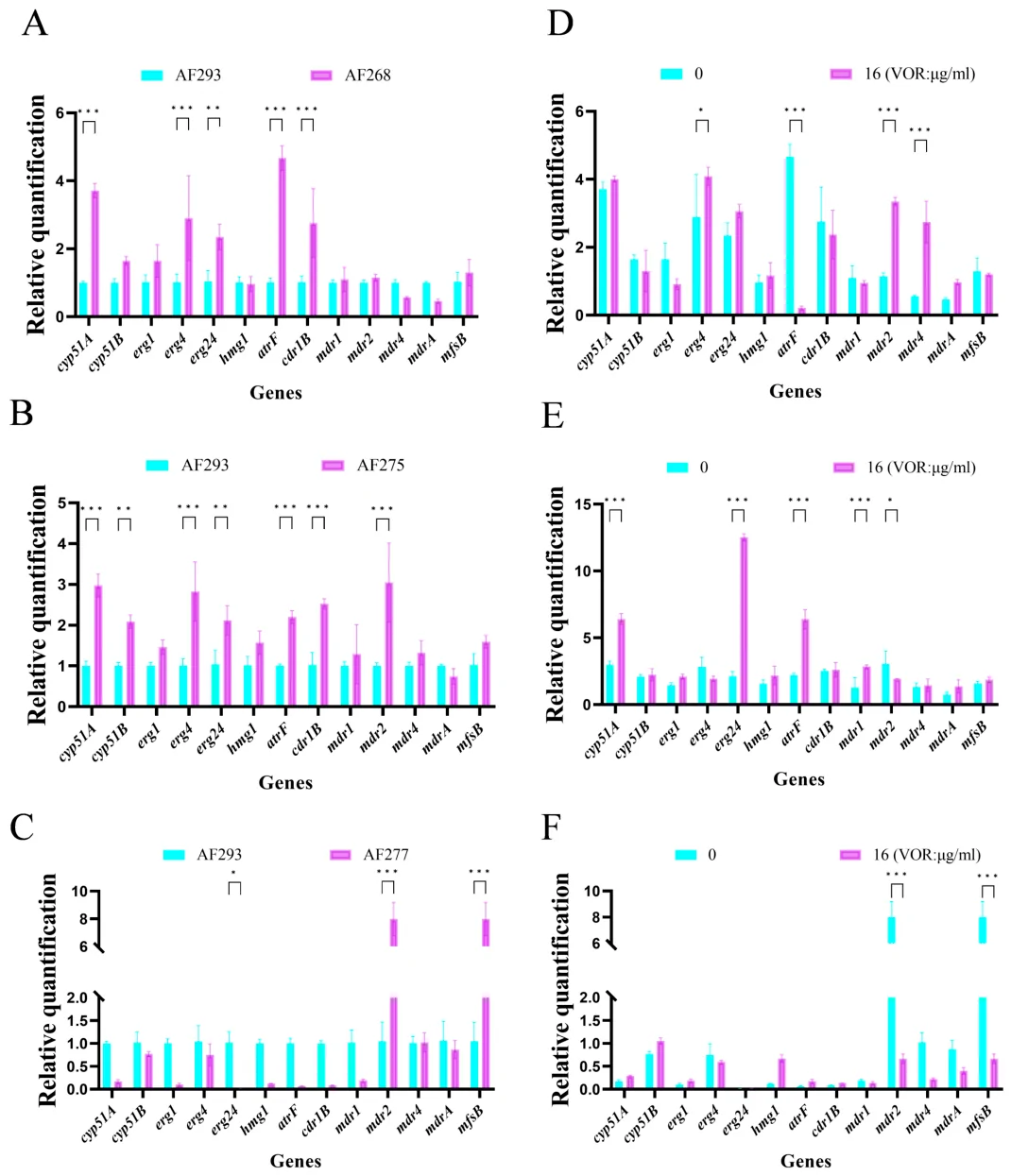
Expression of ergosterol biosynthesis and efflux pump-associated genes. (**A**–**C**) Basal expression in AF268, AF275, and AF277, respectively, relative to AF293 after 24 h of culture. (**D**–**F**) Expression in AF268, AF275, and AF277, respectively, after exposure to 16 µg/mL VRC for 8 h relative to the corresponding untreated culture. For each panel, individual genes were analyzed using multiple unpaired two-tailed Student’s *t*-tests with Holm–Šidák correction for multiple comparisons. * *p* < 0.05, ** *p* < 0.01, *** *p* < 0.001.

RT-qPCR was used to measure the expression of ergosterol biosynthesis genes (*cyp51A*, *cyp51B*, *erg1*, *erg4*, *erg24*, and *hmg1*) and efflux pump-associated genes (*atrF*, *cdr1B*, *mdr1*, *mdr2*, *mdr4*, *mdrA*, and *mfsB*). Basal expression in each clinical isolate was compared with that in AF293, whereas expression after exposure to 16 µg/mL VRC for 8 h was compared with that in the corresponding untreated control. AF268 showed significantly higher basal expression of *cyp51A*, *cyp51B*, and *atrF* and lower expression of *mdr4* and *mdrA* than AF293 (Figure 6A). VRC exposure significantly increased *mdr2*, *mdr4*, and *mdrA* expression and decreased *atrF* expression in AF268 (Figure 6D). In AF275, basal expression of *cyp51A*, *cyp51B*, *atrF*, and *cdr1B* was significantly higher than in AF293 (Figure 6B), and VRC exposure significantly increased *cyp51A*, *erg24*, and *atrF* expression (Figure 6E). AF277 showed significantly lower basal expression of *cyp51A*, *erg1*, *erg24*, *hmg1*, *atrF*, *cdr1B*, and *mdr1* but higher expression of *mdr2* and *mfsB* than AF293 (Figure 6C). Following VRC exposure, *cyp51A*, *cyp51B*, *hmg1*, and *cdr1B* expression significantly increased, whereas *mdr2*, *mdr4*, and *mfsB* expression significantly decreased in AF277 (Figure 6F).

## 4. Discussion

Invasive aspergillosis caused by *A. fumigatus* remains a major clinical challenge, and VRC and isavuconazole are recommended as first-line agents [14]. Triazole resistance complicates treatment and is associated with delayed effective therapy and increased mortality [36,37]. In this study, three of 22 clinical isolates (AF268, AF275, AF277) exhibited high-level VRC resistance. Despite sharing the canonical *cyp51A* TR46/Y121F/T289A genotype, the three clinical isolates showed phenotypic and transcriptional differences relative to AF293.

Conidia are the principal propagules responsible for environmental dissemination and the initiation of *A. fumigatus* infection. Owing to their small size, inhaled conidia can reach the lower respiratory tract, where insufficient host clearance permits germination and hyphal growth [2]. Conidiation is therefore an important trait related to fungal dissemination and fitness [38]. All three VRC-resistant isolates produced fewer conidia than AF293 at 72 h; however, at 120 h, AF268 exceeded AF293, whereas AF275 remained significantly less productive at both time points (Figure 3A,B). These results indicate isolate-specific conidiation patterns relative to AF293 despite the shared *cyp51A* TR46/Y121F/T289A genotype, suggesting that this resistance background does not necessarily correspond to a uniform conidiation phenotype.

Biofilm formation is another important feature of *A. fumigatus* biology and contributes to persistent infection and reduced antifungal susceptibility [39,40]. Extracellular-matrix production, local hypoxia, and metabolic alterations within biofilms can enhance tolerance to antifungal agents, including azoles [39,40]. However, the relationship between biofilm formation and acquired azole resistance varies among isolates and may depend on strain background and resistance mechanism [39,40]. In our study, neither XTT-based metabolic activity nor crystal-violet biomass differed significantly between any of the three clinical isolates and AF293 (Figure 3C,D), suggesting biofilm formation was not associated with the high-level VRC-resistant phenotype of these isolates under the conditions tested.

Virulence in *A. fumigatus* is multifactorial and depends on fungal growth, stress adaptation, immune interactions, and host damage [38,41]. *G. mellonella* is widely used as a comparative model for assessing strain-dependent virulence and fungal fitness [34,42]. In this study, larvae infected with AF268, AF275, or AF277 survived significantly longer and carried lower fungal burdens than larvae infected with AF293, indicating attenuated virulence of the three clinical isolates relative to AF293 in this model. Previous work has likewise demonstrated substantial strain- and model-dependent variation in *A. fumigatus* virulence, including among azole-resistant isolates [43,44]. Taken together, these observations indicate that high-level VRC resistance does not necessarily coincide with increased virulence and that the resistance and virulence phenotypes can vary independently across strain backgrounds.

Lanosterol 14α-demethylase (Cyp51) is the primary molecular target of triazoles and a key enzyme in ergosterol biosynthesis [18,45]. Among the established *cyp51A*-mediated resistance mechanisms, the TR46/Y121F/T289A genotype is strongly associated with reduced VRC susceptibility, while isolates carrying this genotype may show variable susceptibility to other azoles [25]. Consistent with these observations, AF268, AF275, and AF277 all carried TR46/Y121F/T289A and exhibited VRC MICs of 32 µg/mL and ISA MICs of >16 µg/mL, while retaining relatively low ITR and POS MICs. The additional Cyp51A amino acid differences Y46F, V172M, T248N, E255D, and K427E were not interpreted as independent resistance determinants, because these substitutions have also been reported in azole-susceptible *A. fumigatus* backgrounds and do not consistently confer reduced triazole susceptibility [46,47]. Although alterations in *cyp51B* and *hmg1* can contribute to triazole resistance in some genetic backgrounds, their relevance should be evaluated in the context of the specific variant. Cyp51B mutations or overexpression have been experimentally shown to reduce triazole susceptibility [48], but no Cyp51B amino acid substitutions were detected in the three isolates examined here. All three isolates carried the Hmg1 substitutions S212P and Y564F. Importantly, both positions lie outside the predicted Hmg1 sterol-sensing domain, whereas current functional evidence most strongly supports resistance-associated Hmg1 alterations affecting the sterol-sensing region [49,50]. Thus, the contribution of S212P and Y564F to the resistant phenotype cannot be established from the present sequence data and requires functional validation. At the transcriptional level, the three isolates showed distinct expression patterns. AF268 showed increased basal expression of *cyp51A*, *cyp51B*, and *atrF*, together with reduced *mdr4* and *mdrA* expression, whereas AF275 showed increased expression of *cyp51A*, *cyp51B*, *atrF*, and *cdr1B*. AF277 displayed a contrasting basal profile, with reduced expression of several ergosterol biosynthesis and efflux-associated genes but increased *mdr2* and *mfsB* expression. VRC exposure further elicited isolate-specific transcriptional responses. The coordinated regulation of *cyp51A* and *cdr1B* by AtrR provides a well-established link between target-gene expression and drug efflux in *A. fumigatus* [20]. Moreover, azole exposure is known to induce extensive transcriptional responses involving ergosterol biosynthesis and transporter genes [23,51]. Together, these findings indicate that isolates sharing the TR46/Y121F/T289A genotype can show differences in basal gene expression compared with AF293 and in their responses to VRC compared with their own untreated controls. These expression differences should therefore be regarded as resistance-associated, strain-specific responses rather than direct evidence that individual differentially expressed genes independently confer resistance.

Several limitations should be acknowledged. First, only three VRC-resistant isolates were characterized in depth, and most isolates in the collection originated from respiratory specimens; the present data therefore cannot be used to estimate regional prevalence, transmission, or population-level genotype frequencies. Second, AF293 was the principal comparator for phenotypic and transcriptional analyses. Although AF293 is a widely used reference strain, it is not a matched azole-susceptible clinical isolate from the same collection, limiting our ability to distinguish resistance-associated features from strain-background effects. Inclusion of additional molecularly confirmed azole-susceptible clinical *A. fumigatus* isolates would help address this limitation. Third, sequencing was restricted to selected resistance-associated loci, and whole-genome sequencing would be required to define broader genetic backgrounds, relatedness among AF268, AF275, and AF277, and potential additional variants. Fourth, the Hmg1 variants and the observed transcriptional changes were not functionally validated, so their causal contribution to VRC resistance cannot be established. Finally, the attenuated virulence observed in *G. mellonella* is model-dependent and should be validated using additional clinical comparators and, where appropriate, mammalian infection models.

Overall, these findings extend the phenotypic characterization of clinical *A. fumigatus* isolates carrying *cyp51A* TR46/Y121F/T289A and highlight isolate-specific phenotypic and transcriptional features observed relative to AF293. These features warrant broader genomic and functional investigation, but they should not be considered independent resistance mechanisms without direct validation.

## 5. Conclusions

In summary, the three clinical *A. fumigatus* isolates with high-level VRC resistance shared the canonical *cyp51A* TR46/Y121F/T289A genotype and exhibited isolate-specific patterns of conidiation, virulence in *G. mellonella*, and basal and VRC-induced expression of ergosterol biosynthesis and efflux pump genes. No Cyp51B amino acid substitutions were detected, and the shared Hmg1 S212P/Y564F variants should be considered of uncertain functional significance in the absence of genetic validation. The high VRC MICs observed in AF268, AF275, and AF277 are consistent with the established phenotype of TR46/Y121F/T289A. The transcriptional differences identified here may reflect strain-specific adaptive responses, but their contribution to resistance requires direct functional testing. Larger collections, whole-genome sequencing, and allele-replacement or complementation studies will be necessary to determine how genetic background and transcriptional regulation influence the phenotypic characteristics of TR46/Y121F/T289A *A. fumigatus* isolates.

## Figures and Tables

**Table 1:** Broth microdilution results for 22 clinical filamentous fungal isolates.

Strain	ITR	VRC	AmB	FLC
AF220	<0.25	<0.25	<0.50	>64
AF221	<0.50	<1.00	<0.50	>128
AF222	<0.25	<0.25	<0.50	>128
AF224	<1.00	<1.00	<0.50	>128
AF230	<1.00	<0.25	<0.50	>128
AF231	<2.00	<0.50	<1.00	>128
AF234	<1.00	<0.50	<0.50	>128
AF235	<1.00	<1.00	<1.00	>128
AF248	<0.25	<1.00	<0.50	>128
AF249	<0.50	<0.50	<0.50	>128
AF253	<0.50	<0.50	<0.25	>128
AF261	<0.50	<0.50	<0.50	>128
AF264	<0.50	<0.50	<0.50	>128
AF266	<0.50	<0.25	<0.50	>128
AF268	<0.50	32	<0.50	>128
AF271	<1.00	<1.00	<0.50	>128
AF273	<0.50	<0.25	<0.50	>128
AF274	<0.25	<0.50	<0.50	>128
AF275	<0.50	32	<0.50	>128
AF276	<0.50	<0.50	<0.50	>128
AF277	<1.00	32	<0.50	>128
AF280	<0.25	<0.50	<0.50	>128

AmB, amphotericin B; VRC, voriconazole; ITR, itraconazole; FLC, fluconazole; MIC, minimum inhibitory concentration. Values are expressed as MICs (µg/mL).

**Table 2:** Broth microdilution results for 3 clinically resistant strains.

Strain	AmB	VRC	ITR	POS	ISA	FLC	MF	ANI
AF268	<0.5	32	<0.5	0.5	>16	>128	<0.0625	<0.0625
AF275	<0.5	32	<0.5	0.25	>16	>128	<0.0625	<0.0625
AF277	<0.5	32	<1.0	0.5	>16	>128	0.0625	0.0625

AmB, amphotericin B; VRC, voriconazole; ITR, itraconazole; POS, posaconazole; ISA, isavuconazole; FLC, fluconazole; MF, micafungin; ANI, anidulafungin. Values are expressed as MICs (µg/mL), except for MF and ANI, which are expressed as MECs (µg/mL).

## Data Availability

The datasets used and analyzed during the current study are available from the corresponding author on reasonable request.

## Supplementary Materials

The following supporting information can be downloaded at: https://www.mdpi.com/article/10.3390/pathogens15101078/s1, Figure S1: Multiple sequence alignment of Cyp51A amino acid sequences from AF293 and the clinical isolates AF268, AF275, and AF277. Table S1: Patient demographics and specimen sources of clinical isolates; Table S2: Primers used for PCR and RT–qPCR assays.
