# Supplementary material for: Phenotypic and Molecular Characterization of Three Clinical Voriconazole-Resistant Aspergillus fumigatus Isolates Harboring the cyp51A TR46/Y121F/T289A Genotype
Source: Pathogens. 2026 Oct 13;15(10):1078. doi: 10.3390/pathogens15101078 (PMC13708100; doi:10.3390/pathogens15101078)
Supplement: Supplementary file 1 [file pathogens-15-01078-s001.zip › pathogens-4565823-supplementary.pdf]

Supplementary Table S1. Patient demographics and specimen sources of clinical isolates

Clinical isolate ID	Patient age (years)	Patient sex	Specimen type
AF220	80	Male	Sputum
AF221	57	Male	Sputum
AF222	77	Male	Sputum
AF224	53	Female	Sputum
AF230	57	Male	Sputum
AF231	72	Male	Sputum
AF234	67	Male	Sputum
AF235	55	Male	Sputum
AF248	83	Male	Sputum
AF249	86	Female	Sputum
AF253	68	Male	Sputum
AF261	76	Male	Bronchoalveolar lavage fluid
AF264	67	Male	Bronchoalveolar lavage fluid
AF266	72	Male	Sputum
AF268	86	Male	Sputum
AF271	76	Male	Sputum
AF273	74	Male	Bronchoalveolar lavage fluid
AF274	61	Male	Sputum
AF275	67	Female	Sputum
AF276	85	Male	Sputum
AF277	71	Female	Sputum
AF280	68	Male	Sputum

Supplementary Table S2. Primers used for PCR and RT–qPCR assays

Gene	Experiment	Forward Primer	Reverse Primer
cyp51A	PCR	CTTTAGATTCGGTGGACGCA	GGAGACTAGCACAAAGGGCATA
cyp51B	PCR	GCGAGACACTCAGCCTACCTTTAT	GCAAGTCTATCAAGAAGAGGCATG
hmg1	PCR	GTGTCTCACCCCTGCTAACCTTCAA	AGGCTCGGCTACATTCCTCTCTTGG

Gene	Experiment	Forward Primer	Reverse Primer
ITS	PCR	TCCGTAGGTGAACCTGCGG	TCCTCCGCTTATTGATATGC
β-tubulin (*benA*)	PCR	GGTAACCAAATCGGTGCTGCTTTC	ACCCTCAGTGTAGTGACCCTTGGC
Afu1g10910	RT-qPCR	TTCCCAACAACATCCAGACC	CGACGGAACATAGCAGTGAA
cyp51A	RT-qPCR	TCCAAGCTGATGGAGCAGAA	GTGAATCGCGCAGATAGTCC
cyp51B	RT-qPCR	GCGGGTCAACATTCTTCCTC	GAGGCAAGTCAGATCCGAGA
erg1	RT-qPCR	CAAGCAACATCACCCGCATA	TTCGGGAATGTCAACGAGGA
erg4	RT-qPCR	CATGTGACGTGTTCTTCGCT	ACCCGACGCTCATATTCCAT
erg24	RT-qPCR	TTTAGCCCTGGGCACATACA	CTGGTGCCAATTCTCTCAGC
hmg1	RT-qPCR	ACCACCATCCTCTGCATCAA	ATTCGGTCAGAGGCCAATCA
atrF	RT-qPCR	AGTCCAGCTACACGGTTTCA	TGTCGAGGGATTCACCGTAG
cdr1B	RT-qPCR	GCCTCGACAGTCAAACTTCC	GTCGAATCGTTGGAACAGCA
mdr1	RT-qPCR	CCCTGAGCTTGGTTACATGC	GCTAATTGCCTTGGCGTACA
mdr2	RT-qPCR	ATTGGAGCCAAAGTCGAACG	CTCGATGTCCTCATGCTCCT
mdr4	RT-qPCR	GGTATCGTCTTCGGTGACCT	CACTCACCAAACACCAGCAA
mdrA	RT-qPCR	GTCCAAGTTCAAGCCAAGCA	GACGGGTAATTGGTCCAAGC
mfsB	RT-qPCR	TACCTGGCACTCATCAGCAA	TCGAACCGAGAACCTTGGAA

Supplementary Figure S1.

A

```
CYP51A-AF293.seq    FHWVPYLGSTISYGIDPYKFFFACREKYGDIFTFILLGQK    80
CYP51A-AF275.seq    FHWVPFLGSTISYGIDPYKFFFACREKYGDIFTFILLGQK    80
CYP51A-AF268.seq    FHWVPFLGSTISYGIDPYKFFFACREKYGDIFTFILLGQK    80
CPY51A-AF277.seq    FHWVPFLGSTISYGIDPYKFFFACREKYGDIFTFILLGQK    80
Consensus           fhwvp lgstisygidpykfffacrekygdiftfillgqk

CYP51A-AF293.seq    TTVYLGVQGNEFILNGKLKDVNAEEVYSPLTTPVFGSDVV    120
CYP51A-AF275.seq    TTVYLGVQGNEFILNGKLKDVNAEEVYSPLTTPVFGSDVV    120
CYP51A-AF268.seq    TTVYLGVQGNEFILNGKLKDVNAEEVYSPLTTPVFGSDVV    120
CPY51A-AF277.seq    TTVYLGVQGNEFILNGKLKDVNAEEVYSPLTTPVFGSDVV    120
Consensus           ttvylgvqgnefilngklkdvnaeevyspltTpvfgsdvv

CYP51A-AF293.seq    YDCPNSKLMEQKKFIKYGLTQSALESHVPLIEKEVLDYLR    160
CYP51A-AF275.seq    FDCPNSKLMEQKKFIKYGLTQSALESHVPLIEKEVLDYLR    160
CYP51A-AF268.seq    FDCPNSKLMEQKKFIKYGLTQSALESHVPLIEKEVLDYLR    160
CPY51A-AF277.seq    FDCPNSKLMEQKKFIKYGLTQSALESHVPLIEKEVLDYLR    160
Consensus            dcpnsklmeqkkfikygltqsaleshvpliekevldylr

CYP51A-AF293.seq    DSPNFQGSSGRVDISAAMAEITIFTAARALQGQEVRSKLT    200
CYP51A-AF275.seq    DSPNFQGSSGRMDISAAMAEITIFTAARALQGQEVRSKLT    200
CYP51A-AF268.seq    DSPNFQGSSGRMDISAAMAEITIFTAARALQGQEVRSKLT    200
CPY51A-AF277.seq    DSPNFQGSSGRMDISAAMAEITIFTAARALQGQEVRSKLT    200
Consensus           dspnfqgssgr disaamaeitiftaaralqgqevrsklt

CYP51A-AF293.seq    AEFADLYHDLDKGFTPINFMLPWAPLPHNKKRDAAHARMR    240
CYP51A-AF275.seq    AEFADLYHDLDKGFTPINFMLPWAPLPHNKKRDAAHARMR    240
CYP51A-AF268.seq    AEFADLYHDLDKGFTPINFMLPWAPLPHNKKRDAAHARMR    240
CPY51A-AF277.seq    AEFADLYHDLDKGFTPINFMLPWAPLPHNKKRDAAHARMR    240
Consensus           aefadlyhdldkgftpinfmlpwaplphnkkrdaaharmr

CYP51A-AF293.seq    SIYVDIITQRRLDGEKDSQKSDMIWNLMNCTYKNGQQVPD    280
CYP51A-AF275.seq    SIYVDIINQRRLDGDKDSQKSDMIWNLMNCTYKNGQQVPD    280
CYP51A-AF268.seq    SIYVDIINQRRLDGDKDSQKSDMIWNLMNCTYKNGQQVPD    280
CPY51A-AF277.seq    SIYVDIINQRRLDGDKDSQKSDMIWNLMNCTYKNGQQVPD    280
Consensus           siyvdii qrrldg kdsqksdmiwnlmnctykngqqvpd

CYP51A-AF293.seq    KEIAHMMITLLMAGQHSSSSISAWIMLRLASQPKVLEELY    320
CYP51A-AF275.seq    KEIAHMMIALLMAGQHSSSSISAWIMLRLASQPKVLEELY    320
CYP51A-AF268.seq    KEIAHMMIALLMAGQHSSSSISAWIMLRLASQPKVLEELY    320
CPY51A-AF277.seq    KEIAHMMIALLMAGQHSSSSISAWIMLRLASQPKVLEELY    320
Consensus           keiahmmi llmagqhssssisawimlrlasqpkvleely

CYP51A-AF293.seq    QEQLANLGPAGPDGSLPPLQYKDLDKLPFHQHVIRETLRI    360
CYP51A-AF275.seq    QEQLANLGPAGPDGSLPPLQYKDLDKLPFHQHVIRETLRI    360
CYP51A-AF268.seq    QEQLANLGPAGPDGSLPPLQYKDLDKLPFHQHVIRETLRI    360
CPY51A-AF277.seq    QEQLANLGPAGPDGSLPPLQYKDLDKLPFHQHVIRETLRI    360
Consensus           qeqlanlgpagpdgslpplqykdldklpfhqhviretlri

CYP51A-AF293.seq    HSSIHSIMRKVKSPLPVPGTPYMIPPGRVLLASPGVTALS    400
CYP51A-AF275.seq    HSSIHSIMRKVKSPLPVPGTPYMIPPGRVLLASPGVTALS    400
CYP51A-AF268.seq    HSSIHSIMRKVKSPLPVPGTPYMIPPGRVLLASPGVTALS    400
CPY51A-AF277.seq    HSSIHSIMRKVKSPLPVPGTPYMIPPGRVLLASPGVTALS    400
Consensus           hssihsimrkvksplpvpgtpymippgrvllaspgvtals

CYP51A-AF293.seq    DEHFPNAGCWDPHRWENQATKEQENDKVVDYGYGAVSKGT    440
CYP51A-AF275.seq    DEHFPNAGCWDPHRWENQATKEQENDEVVDYGYGAVSKGT    440
CYP51A-AF268.seq    DEHFPNAGCWDPHRWENQATKEQENDEVVDYGYGAVSKGT    440
CPY51A-AF277.seq    DEHFPNAGCWDPHRWENQATKEQENDEVVDYGYGAVSKGT    440
Consensus           dehfpnagcwdphrwenqatkeqend vvdygygavskgt
```

Supplementary Figure S1. Multiple sequence alignment of Cyp51A amino acid sequences from AF293 and the clinical isolates AF268, AF275, and AF277.
